# Development of a Reduced‐Volume Acute Lethality Toxicity Test for *Hyalella azteca*

**DOI:** 10.1002/etc.4840

**Published:** 2020-09-16

**Authors:** Maegan R. Rodrigues, Richard A. Frank, Daniel M. Schissler, Lorna E. Deeth, Lisa R. Brown, Amanda M. Hedges, D. George Dixon, L. Mark Hewitt, Adrienne J. Bartlett

**Affiliations:** ^1^ Department of Biology University of Waterloo Waterloo Ontario Canada; ^2^ Water Science and Technology Directorate Environment and Climate Change Canada Burlington Ontario Canada; ^3^ Department of Mathematics and Statistics University of Guelph Guelph Ontario Canada

**Keywords:** Freshwater toxicology, Mixture toxicology, Invertebrate toxicology, Amphipod, Method development, Effects‐directed analysis

## Abstract

Effects‐directed analysis (EDA) is used to identify the principal toxic components within a complex mixture using iterative steps of chemical fractionation guided by bioassay results. Bioassay selection can be limited in EDA because of the volume requirements for many standardized test methods, and therefore, a reduced‐volume acute toxicity test that also provides whole‐organism responses is beneficial. To address this need, a static, 7‐d, water‐only, reduced‐volume method (50 mL, 10 organisms) was developed for *Hyalella azteca* that substantially decreases the volume requirements of standard‐volume acute test exposures (200–500 mL of test solution, 15–20 organisms) while maintaining water quality and meeting control survival criteria. Standard‐ and reduced‐volume methods were compared by conducting concurrent toxicity tests with 2 inorganic toxicants (KCl and CdCl_2_) and 2 organic mixtures of naphthenic acid fraction components (NAFCs) to evaluate test performance. There was no difference between methods when comparing the median lethal concentrations (LC50s) for KCl and both NAFC mixtures (*p* > 0.05). The LC50s for CdCl_2_ were statistically different (*p* = 0.0002); however, this was not considered biologically meaningful because the difference between LC50s was <2‐fold. In conclusion, the reduced‐volume *H. azteca* test method generated results comparable to standard‐volume test methods and is suitable for use in situations where limited testing material is available, such as when conducting EDA. *Environ Toxicol Chem* 2020;39:2221–2227. © 2020 Her Majesty the Queen in Right of Canada. Environmental Toxicology and Chemistry published by Wiley Periodicals LLC on behalf of SETAC. Reproduced with the permission of the Minister of Environment and Climate Change Canada.

## INTRODUCTION

Effects‐directed analysis (EDA) is a tool used to determine the drivers of toxicity within complex mixtures through the use of chemical and biological techniques (Brack et al. 2016). This type of analysis utilizes a series of iterative steps of chemical fractionation guided by bioassay results to identify the principal toxic components within complex mixtures (Brack et al. 2016; Altenburger et al. 2019). However, the toxicological analysis of complex mixtures is often difficult to interpret, and the toxic drivers within the mixture remain unknown. For example, an individual compound, a class of compounds, or interactions (additive and nonadditive) between mixture components could cause toxicity. Due to the complexity of environmental mixtures, multiple phases of EDA and biological testing are often required before the toxic components in a mixture can be determined.

Each step of chemical fractionation and biological testing during the course of the EDA results in consumption of the stock extract of interest; thus, decreased quantities of the testing solutions are available with each successive phase of EDA. For this reason, the number of measurable endpoints and the choice of a bioassay are restricted to tests that utilize minimal volumes. Ideally, bioassay selection is based on environmental relevance and organism sensitivities, but practical volume requirements must also be taken into consideration. For example, the marine bacterium *Vibrio fischeri* (Microtox^®^ assay) requires <10 mL of solution to complete a standard 15‐min bioluminescence exposure (Azur Environmental 1995), whereas the 96‐h rainbow trout standard acute toxicity test requires a minimum of 6 L of test solution (Environment Canada 1990). Consequently, *V. fischeri* is commonly used as a test organism for EDA with comparable results to more complex, freshwater organisms (Doherty 2001; Clemente and Fedorak 2005), despite not always being the most sensitive species (Bartlett et al. 2017). The selection of in vitro and in vivo biological assays for use in EDA has been trending toward high‐throughput methods such as miniaturized assays or in vitro assays which require minimal volumes (Burgess et al. 2013). In vitro biological assays are common in EDA because of the specificity of endpoints (i.e., mutagenicity, genotoxicity, etc.) but generally do not have the ability to address the bioavailability of toxicants and may not be as ecologically relevant as in vivo assays (Burgess et al. 2013), requiring additional exposures to validate whole‐organism effects. Toxicity identification evaluation (TIE) is a method similar to EDA in that it utilizes whole‐organism toxicity tests to characterize and identify toxicant classes in environmental samples. However, the TIE approach lacks specificity for toxicant identification when compared with the fractionation approach of EDA. The addition of whole‐organism toxicity tests to EDA will enhance the ecological relevance of the endpoint and provide a fuller understanding of the bioavailability of the identified toxicants. Unfortunately, due to the consumptive nature of bioassay testing, the amount of test material available within a given point in an EDA investigation is a function of the quantity initially recovered and the number of fractionation steps completed. It is therefore important, wherever possible, to utilize whole‐organism assays that consume minimal solution volumes.

*Hyalella azteca*, a freshwater amphipod, is commonly found in many aquatic environments, such as temperate lakes, ponds, slow‐flowing streams, and rivers, within North America. This species is widely used in toxicity testing; it is sensitive to many contaminants, including metals and organics (Borgmann and Munawar 1989; Schubauer‐Berigan et al. 1993; Phipps et al. 1995; Borgmann et al. 2005; Bauer et al. 2019); and standard methods have been developed for its use in water‐only and sediment tests (US Environmental Protection Agency 2000; Environment and Climate Change Canada 2017). Studies have shown that *H. azteca* is actually a cryptic species complex, consisting of many genetically distinct species, and that laboratory cultures in Canada and the United States generally consist of 2 clades (Major et al. 2013; Leung et al. 2016). Therefore, the origin of amphipods used in toxicity testing and potential differences in sensitivity to contaminants should be considered when comparing data in the scientific literature. Because of its ubiquity and abundance, lower trophic level in aquatic food webs, and sensitivity to a wide variety of chemicals, *H. azteca* is an ideal organism for toxicity testing of environmental mixtures. Recent research has demonstrated that *H. azteca* is more sensitive to components of oil sands process–affected water than other commonly used invertebrate species such as *V. fischeri*, *Ceriodaphnia dubia*, and *Daphnia magna* (Bartlett et al. 2017; Bauer et al. 2019). However, the volume requirements of standardized methods for *H. azteca* can be prohibitive for use in EDA. Currently, standardized (14‐d) water‐only *H. azteca* test methods recommend using 275 mL and 10 organisms per test replicate and 5 or more replicates per test concentration (Environment and Climate Change Canada 2017). Standard‐volume acute (7‐d) water‐only toxicity tests routinely conducted in our research laboratory use 200 to 500 mL and 15 to 20 organisms per test replicate, with 4 to 6 replicates per test concentration (Table 1; Borgmann et al. 2005; Bartlett et al. 2017). Therefore, development of a reduced‐volume acute test for *H. azteca* would be beneficial for EDA research intent on using this organism.

**Table 1:** Comparison of standard‐volume and reduced‐volume methods used in acute *Hyalella azteca* toxicity tests

Method	Volume (mL)	No. of organisms	Test length (days)	Replicates per treatment	Endpoint	Fed
Borgmann et al. (2005); standard volume	400	15	7	2–5	Survival	2.5 mg, days 0 and 4
Bartlett et al. (2017); standard volume	500	20	7	4–6	Survival	2.5 mg, days 0 and 4
Bartlett et al. (2017); standard volume	200	15	7	4–6	Survival	2.5 mg, days 0 and 4
Environment and Climate Change Canada (2017); standard volume	275	10	14	≥5	Survival, growth	6.3 mg, 3 times per week (nonconsecutive days)
Present study; reduced volume	50	10	7	6–9	Survival	0.3 mg, days 0 and 4

The objective of the present study was to develop and validate an acute (7‐d, survival), reduced‐volume, water‐only test method with *H. azteca* that can be used in situations where the availability of test solutions is limited, such as EDA. Two important considerations during this process were 1) maintaining adequate water quality, particularly dissolved oxygen and ammonia, and 2) meeting recommended control survival criteria (Environment and Climate Change Canada 2017). To assess the validity of this reduced‐volume test method*,* parallel tests were simultaneously conducted using a variation of standard‐volume methods routinely used in our laboratory (200–500 mL, 15–20 amphipods per replicate; Borgmann et al. 2005; Bartlett et al. 2017) and a 4‐ to 10‐fold reduced‐volume method (50 mL, 10 amphipods per replicate; Table 1). The 7‐d test duration allowed a direct comparison of the reduced‐volume methods to standard‐volume tests conducted previously in our laboratory, and the number of amphipods per replicate was reduced from 15 to 20 in the standard‐volume tests to 10 in the reduced‐volume tests to lower animal loading density in the test vessels and maintain water quality. We tested 2 inorganic compounds commonly used as reference toxicants (KCl and CdCl_2_) and 2 organic mixtures of naphthenic acid fraction components (NAFCs) previously demonstrated to be toxic to *H. azteca* (Bartlett et al. 2017). The NAFC mixtures were isolated (Frank et al. 2006) from oil sands process–affected water collected from containments at 2 different oil sands mining operations located in Alberta, Canada. The median lethal concentrations (LC50s) from the reduced‐volume acute method were compared with the parallel standard‐volume tests as well as the LC50s reported in the scientific literature to ensure the validity of the reduced‐volume method for use in EDA.

## MATERIALS AND METHODS

*Hyalella azteca* cultures used in the present study originated from a small lake near Burlington, Ontario, Canada (Borgmann et al. 2005), and have been identified as clade 1, which is genetically distinct from most other *H. azteca* cultures in Canada and the United States (clade 8; Major et al. 2013; Leung et al. 2016). Culturing methods used for *H. azteca* are described in detail by Borgmann and Munawar (1989). Cultures and experiments were maintained in dechlorinated municipal tap water from Burlington, Ontario, Canada (originating from Lake Ontario; hardness 128.6 mg/L, alkalinity 88.6 mg/L, pH 8.41) at 25 °C with a 16:8‐h light:dark photoperiod. Amphipods were fed finely ground TetraMin^®^ fish food flakes (Tetra). Juveniles were removed from breeding containers weekly for use in toxicity tests to guarantee that the age of the amphipods was 2 to 10 d at test initiation, which is standard procedure for our laboratory and thus directly comparable to previously published data (Borgmann et al. 2005; Bartlett et al. 2017).

### Preparation of inorganic compounds and NAFC mixtures for toxicity testing

Standard‐ and reduced‐volume tests were completed simultaneously in pairs with KCl, CdCl_2_, and NAFCs from 2 sources, fresh tailings from industry A (collected in 2009) and industry B (collected in 2011; referred to hereafter as NAFCs from industry A and industry B). These organic mixtures have been tested using a standard‐volume *H. azteca* acute (7‐d, 500 mL) assay, with reported LC50s of 16.7 and 27.4 mg/L, respectively (Bartlett et al. 2017). Stock concentrations were reported (Marentette et al. 2015) and stored in 0.05 M NaOH at 4 °C in amber glass. Currently, there are no representative reference mixtures for bitumen‐derived NAFCs, and quantitative analyses can vary. Stock solutions were tested for changes in synchronous fluorescence spectroscopy signal intensity and toxicity over time (including in Bartlett et al. 2017), indicating no observable change in chemical or toxicological profile (data not shown); therefore, the same nominal concentrations were used to allow for direct comparisons between the current and previous bioassays. The same stock solution was used for each pair of standard‐ and reduced‐volume tests. A 55% dilution series (28.1, 15.7, 8.99, 5.06, 2.81, 1.57, 0.90, 0 μg Cd/L) was used for CdCl_2_ test solutions, 80% dilution series (640, 510, 410, 330, 260, 200, 160, 0 mg KCl/L) for KCl test solutions, and 50% dilution series (100, 50, 20, 10, 5, 2, 0 mg/L) for NAFC solutions.

Stock solutions of KCl (1 g KCl/L; Sigma‐Aldrich; Chemical Abstracts Service [CAS] 7447‐40‐7) and CdCl_2_ (18.3 mg CdCl_2_/L; Fisher Scientific; CAS 10108‐64‐2) were prepared in deionized water (Milli‐Q^®^; Millipore) 24 h prior to test setup and mixed with culture water 2 h prior to test initiation to create the test dilutions. Industry A and industry B NAFCs were collected, extracted, and purified as described (Frank et al. 2006), then stored at 4 °C until solution preparation. Concentrations of primary NAFC stock solutions were analyzed using liquid chromatography with time‐of‐flight mass spectrometry (Brunswick et al. 2015) and determined to be 2504 mg/L (industry A) and 1243 mg/L (industry B; Bartlett et al. 2017). Primary NAFC stock solutions were stored in amber vials at 4 °C and pH >12; they are considered stable under these conditions and should not degrade during storage.

Stock solutions of the NAFC mixtures (100 mg/L) were prepared in a salt solution of 0.05 M NaOH in culture water, following procedures described in detail (Bartlett et al. 2017). The NAFC solutions and salt control solutions for parallel standard‐ and reduced‐volume tests were prepared 24 h prior to test setup. The pH of these solutions was adjusted to 8.50 (±0.1) using 1 M HCl. At 2 h prior to test initiation, the pH of NAFC and salt control solutions was readjusted to 8.35 (±0.1), and test treatments were prepared by diluting the NAFC solution with salt control solutions to maintain a constant level of salt in all NAFC treatments.

Water quality parameters (dissolved oxygen, conductivity, pH, total ammonia [NH_3_/NH_4_^+^], and chloride) were measured at test initiation (*t* = 0 d) and take down (*t* = 7 d).

### Standard‐volume method

Static, 7‐d, water‐only *H. azteca* acute toxicity tests were conducted based on standard‐volume test methods used routinely in our laboratory (Borgmann et al. 2005; Bartlett et al. 2017). High‐density polyethylene beakers (480 mL, internal diameter ∼7.0–8.6 cm [bottom–top]) were used to conduct inorganic KCl and CdCl_2_ tests. Test replicates contained a 5 × 5 cm square of cotton gauze, 400 mL of exposure solution, and 15 juvenile amphipods. Glass beakers (250 mL, internal diameter ∼6.3 cm) were used for organic NAFC tests, and test replicates contained a 2.5 × 2.5 cm square of cotton gauze, 200 mL of exposure solution, and 15 juvenile amphipods. Different volumes were used for inorganic versus organic contaminants so that our results were directly comparable to tests conducted previously in our laboratory (Borgmann et al. 2005; Bartlett et al. 2017). Amphipods were fed 1 mL of a ground TetraMin slurry (15 mg/25 mL culture water; 0.04 mg TetraMin per amphipod) per replicate at test initiation and on day 4; survival was assessed at the end of the 7‐d exposure. The CdCl_2_ and KCl tests were aerated for the entire exposure, but the NAFC tests were not aerated to reduce volatilization of the organic compounds. Three tests were completed for each inorganic compound, with each test consisting of 3 replicates per control and 2 replicates per test concentration (total of 6–9 replicates; i.e., *n* = 6–9). Two tests were completed for each organic mixture, with 3 replicates per control and test concentration per test (total of 6 replicates; i.e., *n* = 6).

### Reduced‐volume method

Glass beakers (50 mL, internal diameter ∼3.8 cm) were used for inorganic and organic NAFC exposures. Test replicates contained a 2.5 × 2.5 cm square of cotton gauze, 50 mL of treatment solution, and 10 juvenile amphipods. Amphipods were fed 1 mL of a ground TetraMin slurry (7.5 mg/25 mL culture water; 0.03 mg TetraMin per amphipod) per replicate at test start and on day 4. A reduced food ration was used relative to the standard‐volume method to keep ammonia levels low. Survival was assessed at the end of the 7‐d exposure. Similar to the standard‐volume method, KCl and CdCl_2_ tests were aerated for the entire exposure, but NAFC tests were not aerated in an attempt to decrease compound volatilization. Three tests were completed for each inorganic compound, with each test consisting of 3 replicates per control and 2 replicates per test concentration (total of 6–9 replicates; i.e., *n* = 6–9). Two tests were completed for each organic mixture, with 3 replicates per control and test concentration per test (total of 6 replicates; i.e., *n* = 6).

### Statistical analysis

Data were analyzed using R, Ver 3.3.3 (R Development Core Team 2017); RStudio, Ver 1.0.136 (RStudio Team 2016); and software package *drc* (Ritz et al. 2016). Data were fit to a 4‐parameter log‐logistic model. The LC50s were calculated from pooled data from replicate tests using NAFC concentrations from Bartlett et al. (2017) and nominal concentrations for KCl and Cd. The reduced‐volume test and standard‐volume test LC50s from the same test compounds/mixtures were compared and considered not significantly different if a ratio of the LC50s, within a 95% confidence interval (CI), was equal to 1 and *p* values were >0.05 (Ritz et al. 2006).

## RESULTS AND DISCUSSION

**Figure 1:**
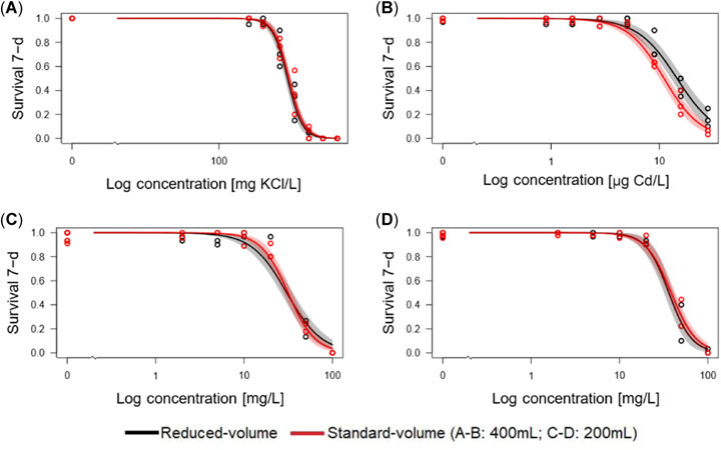
*Hyalella azteca* concentration–response curves at 7 d to 2 inorganic toxicants (potassium chloride [**A**] and cadmium chloride [**B**]) and 2 organic mixtures of naphthenic acid fraction components from oil sands tailings (industry A [**C**] and industry B [**D**]). Gray and pink shadings are the 95% confidence intervals for the reduced‐volume and standard‐volume tests, respectively.

*Hyalella azteca* has been extensively used in toxicity testing due to its widespread abundance in Canadian freshwater environments and its sensitivity to a variety of compounds in both water and sediment exposures (Borgmann and Munawar 1989; Schubauer‐Berigan et al. 1993; Phipps et al. 1995; Borgmann et al. 2005; Bauer et al. 2019). Potassium chloride is a commonly used reference toxicant to monitor the health of laboratory cultures (96‐h LC50 460 mg/L, 95% CI 390–550 mg/L; McNulty et al. 1999), and Cd is one of the most toxic metal anions to *H. azteca* (7‐d LC50 4.41 µg Cd/L, 95% CI 3.47–5.60 µg/L; Borgmann et al. 2005). The differences between the LC50s (Table 2) of the reduced‐volume test and the standard‐volume test were not statistically different for KCl (*p* = 0.34; 293 and 301 mg/L, respectively). The LC50s for KCl were slightly lower than the 96‐h LC50 of 460 mg/L published by McNulty et al. (1999), which could be attributed to differences in test duration (7 d vs 96 h) and clade (clade 1 vs clade 8), but were nonetheless comparable (<2‐fold difference). The difference between the LC50s of the reduced‐volume test and the standard‐volume test for CdCl_2_ was statistically significant (*p* = 0.0002); however, because this difference was <2‐fold (14.5 and 11.0 µg Cd/L, respectively), the difference between test methods was not considered to be biologically meaningful. The model concentration–response curves for standard‐ and reduced‐volume tests partially overlapped within the 95% CI for both reference toxicants (Figure 1). When compared with previous toxicity tests using the same methods (i.e., 400 mL of test solution and 15 amphipods per replicate), the LC50s for CdCl_2_ were higher than those published by Borgmann et al. (2005) but comparable to the range of LC50s generated from reference toxicity tests conducted over the last 15 yr (*n* = 38, mean = 6.66 µg/L, standard deviation = 2.70 µg/L, range = 2.29–13.4 µg/L).

**Table 2:** Comparison of median lethal concentrations (LC50s) from 7‐d *Hyalella azteca* reduced‐volume (50 mL) and standard‐volume (200–400 mL) toxicity tests with KCl, CdCl_2_, and 2 organic mixtures of naphthenic acid fraction components from oil sands tailings (industry A and industry B)

	Treatment							
	CdCl_2_ (μg Cd/L)		KCl (mg/L)		Industry A (mg/L)		Industry B (mg/L)	
Volume of solution per replicate (mL)	50	400	50	400	50	200	50	200
LC50	14.5	11.0	293	301	30.4	31.5	36.2	38.4
Standard error	0.991	0.560	6.25	5.26	2.42	1.82	2.38	2.13
Lower CI	12.6	9.94	281	290	25.7	27.9	31.5	34.3
Upper CI	16.5	12.1	305	311	35.1	35.0	40.9	42.6
Difference (%)	31.8*		2.63		3.49		5.73	
*p*	0.0002*		0.34		0.73		0.48	

Asterisks indicate statistically significant differences between LC50s (*p* < 0.05).

CI = 95% confidence interval.

The effectiveness of the reduced‐volume method was further analyzed using organic solutions (NAFCs) previously determined to be toxic to *H. azteca* (i.e., the 7‐d LC50s for NAFCs from industry A and industry B were reported to be 16.7 and 27.4 mg/L, respectively [Bartlett et al. 2017]). For the present study, the results of the reduced‐volume test were compared with the concurrently completed standard‐volume test for industry A and industry B NAFCs. The differences between the LC50s (Table 2) of the reduced‐volume test and the standard‐volume test were not statistically significant for industry A (*p* = 0.73; 30.4 and 31.5 mg/L, respectively) or industry B (*p* = 0.48; 36.2 and 38.4 mg/L, respectively). In addition, the fit and overlap of the concentration–response models were comparable between methods (Figure 1). The LC50s for industry A and industry B NAFCs were higher than those reported by Bartlett et al. (2017). The concentrations of NAFCs in the stock solutions were not quantified again for the present study, so it is possible that slight changes in chemical composition occurred during storage. However, previous analyses have not indicated changes in the chemical and toxicological profiles of these mixtures over time; and because the differences between the LC50s in the present study and those from Bartlett et al. (2017) were <2‐fold, the bioassay results were comparable between studies.

In addition to achieving comparable toxicity results for inorganic and organic compounds, reduced‐volume tests met recommended criteria for water quality and control performance. Water quality parameters (Tables 3 and 4) were well within acceptable limits (dissolved oxygen >40% or 3.4 mg/L, pH 6.0–8.0 or 0.5 ± control water [Environment and Climate Change Canada 2017], total ammonia <0.3 mM [5.4 mg/L]; [Borgmann 1994]). The NAFC mixtures were not aerated to reduce potential volatilization of organics, and therefore, maintaining adequate water quality was a concern because aeration is occasionally necessary to maintain sufficiently high dissolved oxygen and low ammonia concentrations for optimum amphipod survival. Ammonia concentrations were of particular concern in the reduced‐volume replicates due to the minimal volume of solution per organism combined with the lack of aeration. Although ammonia levels were slightly elevated in reduced‐volume replicates (0.003–0.025 mM, 0.05–0.43 mg/L) in comparison to the full‐volume replicates (0.005–0.018 mM, 0.09–0.31 mg/L), they were still below the recommended concentration range of 0.3 mM (Borgmann 1994). Mean survival of controls in all toxicity tests was 96 to 100%, meeting the 90% test validity criterion recommended for 96‐h water‐only tests (Environment and Climate Change Canada 2017).

**Table 3:** Summary of water quality parameters measured in standard‐volume (400 mL) and reduced‐volume (50 mL) 7‐d exposures of *Hyalella azteca* to solutions of KCl and CdCl_2_

Test compound	Test volume (mL)	Time (days)	Dissolved oxygen (mg/L) Mean (SD)	Specific conductivity (mS/cm) Mean (SD)	pH Mean (SD)	Total ammonia (mM) Mean (SD)	*n* a
KCl	400	0	8.59 (0.24)	0.93 (0.355)	8.35 (0.10)	ND	24
		7	7.62 (0.42)	1.00 (0.596)	8.29 (0.07)	0.005 (0.007)	51
	50	0	8.30 (0.32)	0.94 (0.361)	8.38 (0.09)	ND	24
		7	7.90 (0.65)	0.95 (0.385)	8.30 (0.07)	0.024 (0.036)	51
CdCl_2_	400	0	8.45 (0.41)	0.37 (0.034)	8.32 (0.07)	ND	24
		7	8.52 (0.48)	0.45 (0.042)	8.42 (0.11)	0.018 (0.008)	51
	50	0	8.32 (0.41)	0.37 (0.034)	8.27 (0.06)	ND	24
		7	8.22 (0.30)	0.45 (0.038)	8.33 (0.10)	0.025 (0.023)	51

^a^ *n* is number of measurements.

ND = not determined; SD = standard deviation.

**Table 4:** Summary of water quality parameters measured in standard‐volume (200 mL) and reduced‐volume (50 mL) 7‐d exposures of *Hyalella azteca* to solutions of industry A and industry B naphthenic acid fraction components

Test compound	Test volume (mL)	Time (days)	Dissolved oxygen (mg/L) Mean (SD)	Specific conductivity (mS/cm) Mean (SD)	pH Mean (SD)	Chloride (mg/L) Mean (SD)	Total ammonia (mM) Mean (SD)	*n* a
Industry A	200/50^b^	0	7.53 (0.59)	0.49 (0.081)	8.35 (0.06)	139^b^ (33.1)	ND	16, 11^c^
	200	7	7.44 (0.42)	0.52 (0.064)	8.28 (0.07)	136 (25.7)	0.013 (0.014)	48
	50	7	7.51 (0.32)	0.52 (0.070)	8.26 (0.06)	143 (27.9)	0.012 (0.008)	48
Industry B	200/50^b^	0	8.24 (0.49)	0.47 (0.067)	8.38 (0.07)	119 (24.9)	ND	16
	200	7	8.23 (0.27)	0.46 (0.073)	8.36 (0.03)	122 (26.6)	0.012 (0.009)	48
	50	7	8.04 (0.25)	0.46 (0.072)	8.33 (0.07)	125 (26.2)	0.003 (0.006)	48

^a^ *n* = number of measurements.

^b^ The 0‐d measurements for naphthenic acid fraction components were combined between test methods because of limited availability of test solutions.

^c^ The initial chloride values of controls and stock concentration were not measured for one test; therefore, the number of measurements was lower for that test (*n* = 11).

ND = not determined; SD = standard deviation.

## CONCLUSION

The purpose of the present study was to develop a reduced‐volume, acute, in vivo bioassay method using *H. azteca*, with survival as the endpoint of interest, for use in EDA investigations. Reduced‐volume and standard‐volume tests were completed in parallel using representative inorganic toxicants (KCl, CdCl_2_) and complex mixtures of organic acids isolated from 2 oil sands mining operations. The reduced‐volume method was comparable to the standard‐volume test methods in terms of water quality, control survival, and acute LC50s and lowered volume requirements by 4‐ to 10‐fold. Overall, the development of a reduced‐volume acute method for the *H. azteca* survival bioassay is beneficial for EDA research and other situations in which limited testing materials are available, presenting a viable option for an in vivo test with a sensitive, environmentally relevant, freshwater species.

## Supplemental Data

The Supplemental Data are available on the Wiley Online Library at https://doi.org/10.1002/etc.4840.

## Disclaimer

The views presented in the present study are only held by the authors and are not representative of the official policy of the authors' individual affiliations.

## Author Contribution Statement

M. Rodrigues, D. Schissler, A. Hedges, and L. Brown: experimental concept and design; M. Rodrigues, D. Schissler, and L. Brown: experimental performance; L. Deeth and M. Rodrigues: statistical analysis; M. Rodrigues and A. Bartlett: writing; R. Frank, M. Hewitt, D.G. Dixon, and A. Bartlett: technical and editorial assistance.

## Supporting information

This article includes online‐only Supplemental Data.

Supporting information.

## Data Availability

Data, associated metadata, and calculation tools are available from the corresponding author (adrienne.bartlett@canada.ca).
