# Supplementary material for: Development of a Reduced‐Volume Acute Lethality Toxicity Test for Hyalella azteca
Source: Environ Toxicol Chem. 2020 Sep 16;39(11):2221–7. doi: 10.1002/etc.4840 (PMC7756806; doi:10.1002/etc.4840)
Supplement: Supplementary file 1 — Supporting information. [file ETC-39-2221-s001.docx]

**Table S1:** Summary of percent survival (standard deviation (SD)) in reduced-volume (50 mL) and standard-volume (200 mL, 400 mL) 7-d exposures of *Hyalella azteca* to solutions of KCl, CdCl_2_, and Industry A and B NAFCs (naphthenic acid fraction components). Concentrations of KCl and CdCl_2_ are nominal, and concentrations of NAFCs are measured.

| ***KCl*** |  |  |  |  |  |  |  |  |  |  |  |  |  |  |  |  |
| --- | --- | --- | --- | --- | --- | --- | --- | --- | --- | --- | --- | --- | --- | --- | --- | --- |
| ***Treatment***  ***(mg KCl/L)*** | **Control** | | **160** | | **200** | | **260** | | **328** | | **410** | | **510** | | **640** | |
| ***Test Volume***  ***(mL)*** | 50 | 400 | 50 | 400 | 50 | 400 | 50 | 400 | 50 | 400 | 50 | 400 | 50 | 400 | 50 | 400 |
| ***% Survival*** | 100.0 | 100.0 | 98.3 | 100.0 | 96.7 | 95.6 | 73.3 | 75.6 | 38.0 | 37.8 | 3.3 | 5.6 | 0.0 | 0.0 | 0.0 | 0.0 |
| ***SD*** | 0.0 | 0.0 | 4.1 | 0.0 | 5.2 | 11.7 | 17.5 | 11.7 | 23.2 | 18.7 | 5.2 | 5.0 | 0.0 | 0.0 | 0.0 | 0.0 |
|  |  |  |  |  |  |  |  |  |  |  |  |  |  |  |  |  |
| ***CdCl_2_*** |  |  |  |  |  |  |  |  |  |  |  |  |  |  |  |  |
| ***Treatment***  ***[µg Cd/L]*** | **Control** | | **0.90** | | **1.57** | | **2.81** | | **5.06** | | **8.99** | | **15.7** | | **28.1** | |
| ***Test Volume***  ***(mL)*** | 50 | 400 | 50 | 400 | 50 | 400 | 50 | 400 | 50 | 400 | 50 | 400 | 50 | 400 | 50 | 400 |
| ***% Survival*** | 100.0* | 98.5 | 98.3 | 98.9 | 96.7 | 98.9* | 100.0 | 95.6 | 94.7 | 95.6 | 76.7 | 62.2 | 41.7 | 28.9 | 16.7 | 5.6 |
| ***SD*** | 5.0 | 2.9 | 4.1 | 2.7 | 5.2 | 5.0 | 0.0 | 5.4 | 4.1 | 6.9 | 13.7 | 13.1 | 9.8 | 10.9 | 8.2 | 5.0 |
| **Additional organism found in one replicate; i.e. there were 11 and 16 amphipods in one replicate of control (50 mL volume) and 1.57 µg Cd/L (400 mL volume), respectively, at the end of the tests.* | | | | | | | | | | | | | | | | |
| ***NAFC Industry A*** |  |  |  |  |  |  |  |  |  |  |  |  |  |  |  |  |
| ***Treatment***  ***[mg/L]*** | **Control** | | **Salt Control** | | **2** | | **5** | | **10** | | **20** | | **50** | | **100** | |
| ***Test Volume***  ***(mL)*** | 50 | 200 | 50 | 200 | 50 | 200 | 50 | 200 | 50 | 200 | 50 | 200 | 50 | 200 | 50 | 200 |
| ***% Survival*** | 100.0 | 96.7 | 96.7 | 95.6 | 95.0 | 97.8 | 91.7 | 100.0 | 98.3 | 94.4 | 88.3 | 85.6 | 20.0 | 21.1 | 0.0 | 0.0 |
| ***SD*** | 0 | 5.6 | 5.2 | 6.9 | 5.5 | 3.4 | 4.1 | 0.0 | 4.1 | 10.7 | 11.7 | 10.7 | 12.6 | 6.6 | 0.0 | 0.0 |
|  |  |  |  |  |  |  |  |  |  |  |  |  |  |  |  |  |
| ***NAFC Industry B*** |  |  |  |  |  |  |  |  |  |  |  |  |  |  |  |  |
| ***Treatment***  ***[mg/L]*** | **Control** | | **Salt Control** | | **2** | | **5** | | **10** | | **20** | | **50** | | **100** | |
| ***Test Volume***  ***(mL)*** | 50 | 200 | 50 | 200 | 50 | 200 | 50 | 200 | 50 | 200 | 50 | 200 | 50 | 200 | 50 | 200 |
| ***% Survival*** | 98.3 | 96.7 | 98.3 | 100.0 | 100.0 | 98.9 | 98.3 | 97.8 | 98.3 | 97.8 | 91.7 | 94.4 | 25.0 | 33.3 | 1.7 | 0.0 |
| ***SD*** | 4.1 | 3.7 | 4.1 | 0.0 | 0.0 | 2.7 | 4.1 | 3.4 | 4.1 | 3.4 | 7.5 | 5.0 | 25.9 | 17.9 | 4.1 | 0.0 |
